# Secondary Complications in COVID-19 Patients: A Case Series

**DOI:** 10.3390/tomography8040154

**Published:** 2022-07-15

**Authors:** Maria Paola Belfiore, Gaetano Maria Russo, Luigi Gallo, Umberto Atripaldi, Stefania Tamburrini, Valentina Caliendo, Luigi Impieri, Maria Teresa Del Canto, Giovanni Ciani, Pasquale Parrella, Maria Luisa Mangoni di Santo Stefano, Antonio Alessandro Heliot Salvia, Fabrizio Urraro, Valerio Nardone, Nicola Coppola, Alfonso Reginelli, Salvatore Cappabianca

**Affiliations:** 1 Department of Precision Medicine, University of Campania Luigi Vanvitelli, Piazza Luigi Miraglia, 80138 Naples, Italy; mariapaola.belfiore@unicampania.it (M.P.B.); luigigallo992@gmail.com (L.G.); umbatripaldi@gmail.com (U.A.); vale.caliendo@gmail.com (V.C.); l.impieri1@gmail.com (L.I.); mariateresa.delcanto@unicampania.it (M.T.D.C.); dott.giovanniciani@gmail.com (G.C.); pak.parrella@gmail.com (P.P.); antoniosalvia89@gmail.com (A.A.H.S.); fabrizio.urraro@unicampania.it (F.U.); valerio.nardone@unicampania.it (V.N.); nicola.coppola@unicampania.it (N.C.); alfonso.reginelli@unicampania.it (A.R.); salvatore.cappabianca@unicampania.it (S.C.); 2 Department of Radiology, Ospedale del Mare, Via Enrico Russo, 80147 Naples, Italy; tamburrinistefania@gmail.com; 3 Department of Radiology, Ospedale S. Maria della Pietà, Via della Repubblica, 80035 Nola, Italy; marialuisamangoni@libero.it

**Keywords:** COVID-19, complications, SARS-CoV-2

## Abstract

Introduction. Coronavirus SARS-CoV-2, the causative agent of COVID-19, primarily causes a respiratory tract infection that is not limited to respiratory distress syndrome, but it is also implicated in other body systems. Systemic complications were reported due to an exaggerated inflammatory response, which involves severe alveolar damage in the lungs and exacerbates the hypercoagulation that leads to venous thrombosis, ischemic attack, vascular dysfunction and infarction of visceral abdominal organs. Some complications are related to anticoagulant drugs that are administrated to stabilize hypercoagulability, but increase the risk of bleeding, hematoma and hemorrhage. The aim of this study is to report the diagnostic role of CT in the early diagnosis and management of patients with severe COVID-19 complications through the most interesting cases in our experience. Material and Methods. The retrospective analysis of patients studied for COVID-19 in our institution and hospitals, which are part of the university training network, was performed. Cases. Pneumomediastinum, cortical kidney necrosis, splenic infarction, cerebral ischemic stroke, thrombosis of the lower limb and hematomas are the most major complications that are reviewed in this study. Conclusions. Since the onset of the COVID-19 pandemic, the CT imaging modality with its high sensitivity and specificity remains the preferred imaging choice to diagnose early the different complications associated with COVID-19, such as thrombosis, ischemic stroke, infarction and pneumomediastinum, and their management, which significantly improved the outcomes.

## 1. Introduction

Coronavirus disease 2019 (COVID-19) is a new strain of coronavirus that had not been identified in humans before. Severe acute respiratory syndrome coronavirus 2 (SARS-CoV-2) is an enveloped virus with a positive sense, single-stranded RNA genome with high morbidity and mortality outcomes. Viral upper respiratory tract infections (UR-TIs) represent a common characteristic of this disease, particularly pneumonia [1]. Pa tients with COVID-19 may have a wide range of symptoms, although clinically asympto matic cases can also be found; multiple comorbidities are linked to severe clinical symptoms, such as acute respiratory distress syndrome (ARDS) [2]. The main symptoms of COVID-19 are headache, fever, sore throat, cough, fatigue and breathlessness. Comorbidities in COVID-19 patients can lead to severe clinical manifestation. Additionally, mechanically ventilated patients with severe primary infection complicated by acute respiratory distress syndrome (ARDS) show respiratory symptoms and abnormalities on CT at a 3-month follow-up. Recent studies have reported that, in patients with a more severe primary infection, radiological alterations such as “fibrotic-like” changes in the lung parenchyma persist [3].

SARS-CoV-2 invades host cells through the angiotensin-converting enzyme 2 (ACE2) receptor. Transmembrane protease serine protease-2 (TMPRSS-2) has a crucial role in the virus entry into human cells as well [4]. SARS-CoV-2 entry into host cells stimulates the release as well as activation of monocyte, macrophage, and dendritic cells along with the release of cytokines (interleukins). The infection is accompanied by an aggressive inflammatory response with the release and increase in a large amount of pro-inflammatory cytokines, including interleukins and TNF-α. The excessive production of IL-6 and TNF-α in COVID-19 patients can be linked to a possible cytokine release storm associated with the severity of illness [5]. Dendritic cells may potentially be infected with SARS-CoV-2. Improper activation due to dendritic cell dysfunction can cause T-cell death and fatigue, which may play an important role in COVID-19 immunopathology.

Chest CT plays a prominent role in the diagnosis of COVID-19 disease, demonstrating a great sensitivity in identifying patients with severe lung involvement of viral pneumonia as well as asymptomatic cases. Typical CT features of COVID-19 patients are ground-glass opacities and/or consolidations with subpleural distribution, reticulation/thickened interlobular septa, nodules and lesion distribution, mostly affecting the bilateral lower lobes and posterior segments of the lungs. Chest CT is a useful tool for the detection of COVID-19 and its complications [6]. COVID-19 is known as an infectious disease characterized by pneumonia leading to acute respiratory distress syndrome (ARDS). Patients suffering from SARS-CoV-2 can have multiorgan complications, including cardiac injury, acute respiratory distress syndrome (ARDS), arrhythmias, thromboses, infarcts, bleeding, immune failure, secondary infections and other complications [7].

Cytokine release storm causes a hyperinflammatory and multiorgan disease process that can result in systemic complications in COVID-19-diagnosed patients. Hyperinflammation in SARS-CoV-2 is characterized by an extensive end-organ disease, which is primarily focused the lung and results in acute respiratory distress syndrome (ARDS). The major pathologic feature shown as a result of cytokine storm is hemophagocytosis in lung tissues, which has been previously identified in coronavirus-related severe acute respiratory syndrome (SARS). Therefore, the host immune response due to the activation of the body’s innate immune system probably has a major impact on the disease’s severity and may be fatal because of the body’s antiviral defense mechanisms as well as lung inflammation. Consequently, hyperinflammation and hypercoagulation linked to cytokine release storm are the major cause of hematological, cerebral and respiratory complications in COVID-19 [8].

Among the respiratory complications, pneumomediastinum remains one of the most frequent. The pathophysiological mechanisms of COVID-19-related pneumomediastinum are not yet fully understood.

In addition to the hypothesis of barotrauma or transpulmonary high pressure, some studies hypothesize a remodeling of cartilage during COVID-19 and ARDS, without a complete post-injury regenerative process [9].

In fact, a fibrous-hyaline degeneration of the tracheal rings was found in autoptic series of COVID-19 patients where an alteration of the regular molecular crosstalk involving Wnt5a and SHH during tracheal development and regeneration of adult tissues was appreciated [10,11].

## 2. Objective of This Work

In this study, we report our experience on COVID-19 complications through a series of cases to underline the role of CT in the early diagnosis and management of patients with severe COVID-19 complications. The frequent complications include hematological and respiratory complications. In this paper, we discuss cases presenting the complications of COVID-19.

## 3. Case Series

### 3.1. Case 1: Renal Cortical Necrosis and Splenic Infarction

#### 3.1.1. Clinical History

A 76-year-old female patient exhibited no known comorbidities. She was diagnosed with syncope by the emergency service. Moreover, an oropharyngeal PCR swab was performed and it was positive for COVID-19 pneumonia. Laboratory studies showed leukocytosis (17.8 × 109/L) and neutrophilia (15.6 × 109/L), slightly elevated creatinine (1.14 mg/dL) with an eGFR of 47.1 mL/min/1.73 m2, and markedly elevated D-dimer (7051 µg/L DDU). The patient presented no fever and an oxygen saturation level of 85%.

#### 3.1.2. CT Imaging Findings

**Figure 1:**
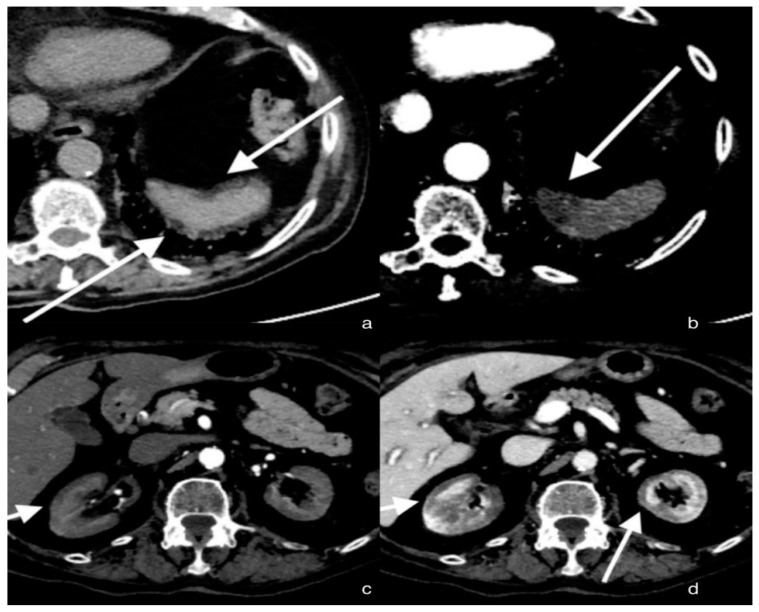
Axial sections of the abdomen in the basal (**a**) and arterial (**b**) phases; the arrows show a small hypo-dense subcapsular area well delimited by the splenic capsule. This area in the arterial phase remains as a hypodense lesion. Lack of enhancement. Axial section of the abdomen in the arterial (**c**) and portal venous (**d**) phases; the arrows show a non-enhancing renal cortex. Images show a normal enhancing renal medulla (acute cortical necrosis), and the hypodense cortex is surrounded by a thin peripheral rim of enhancement in the portal venous phase.

A total-body CT scan was performed after the intravenous administration of a contrast medium under a specific study protocol to assess the aorta and vessels of the lower limb conditions, for the patient’s clinical condition and the elevated D-dimer. The CT scan demonstrated opacification of the left common iliac artery and a filiform left superficial femoral artery. Reduced opacification of the left femoral vein was observed at a later stage of the research. Moreover, a peripheral hypodense flap of both kidneys was observed during the contrast phases, likely as a result of renal cortical necrosis. In addition, a small area of necrotic hypoperfusion, which was located at the upper pole of the spleen, was observed as well. Furthermore, diffuse ground-glass opacity (GGO) can be observed on CT imaging of the lungs, associated with crazy-paving patterns. The total severity score was 18/20 s (according to Chung et al.) [12] (Figure 1).

#### 3.1.3. Discussion

Coronavirus disease 2019 (COVID-19) is characterized by fever, sore throat, cough, dyspnea and different respiratory symptoms in the first stages. Over time, however, it was discovered that this disease can cause systemic damage. Coronavirus SARS-CoV-2 can lead to diffuse alveolar damage (DAD) with severe capillary congestion and variegated consequences affecting not only the lungs but also other organs. This is likely caused by a vascular dysfunction as a result of an exaggerated inflammatory response [13]. The risk of thrombosis is increased in patients suffering from COVID-19 with an incidence of 31% [14]. There are some cases in which signs of necrosis and abdominal viscera infarction have been found, in addition to common pulmonary embolism [15,16]. Cortical kidney necrosis, as well as spleen infarction, are rare but they should be suspected in patients presenting alterations of blood indices related to renal function or in patients suffering left side abdominal pain. An extremely elevated D-dimer is the most significant alteration of coagulation parameters in severely affected COVID-19 patients. This value is recorded more frequently than other coagulation parameters, such as prothrombin time (PT) or aPTT [17]. The increase in the vasoconstrictor angiotensin II, the decrease in the vasodilator angiotensin and the sepsis-induced release of cytokines can cause coagulopathy in COVID-19 patients.

The alteration of the coagulation system, which is triggered by a multisystem inflammation, causes systemic damages. Clinical symptoms of abdominal organ damage are not always easy to evaluate in severely affected patients and the alterations in laboratory tests may be due to multiple causes. The use of ultrasound is certainly useful in a screening process, but emergency conditions often require a rapid and highly reliable diagnosis through a parenchymatous abdominal organs study and the CT scan with a contrast medium is the gold standard in cases of suspected arterial thrombosis.

These findings are more common in patients with an impaired coagulation cascade and a high D-dimer level; in these cases, the findings of organ damage from imaging procedures can resolve concerns about laboratory abnormalities and determine whether organ function is recoverable.

Concerning renal cortical necrosis, contrast-enhanced CT demonstrates a non-enhancing renal cortex and a normal enhancing renal medulla (reverse rim sign) [18]. CT performed during the portal venous phase represents the imaging exam of choice for spleen infarction and typically demonstrates, in the case of peripheral infarction, a wedge-shaped hypo-enhancing region [19].

The diagnosis of thrombosis is often delayed. Early diagnosis and treatment are necessary to prevent infarction of other abdominal organs and to avoid the spreading of this phenomenon to an already affected organ. MDCT plays a crucial role in the early detection of a large vessel and intraparenchymal thrombosis. Timely diagnosis and management of thrombosis can prevent the infarction of parenchymal organs and save part of those already affected.

### 3.2. Case 2: Cerebral Ischemic Stroke

#### 3.2.1. Clinical History

A 60-year-old male patient exhibited no known comorbidities. He was diagnosed with syncope. An oropharyngeal PCR swab was performed and tested positive for COVID-19 pneumonia. A chest CT scan revealed severe lung injury caused by COVID-19 infection and pulmonary embolism. Laboratory studies showed WBC = 7530/μL, lymphocytes (1000/μL), platelets = 328,000/μL, C-reactive protein = 40.76 mg/dL, D-dimer FEU = 6242 ng/mL and fibrinogen = 6.2 g/L.

#### 3.2.2. CT Imaging Findings

**Figure 2:**
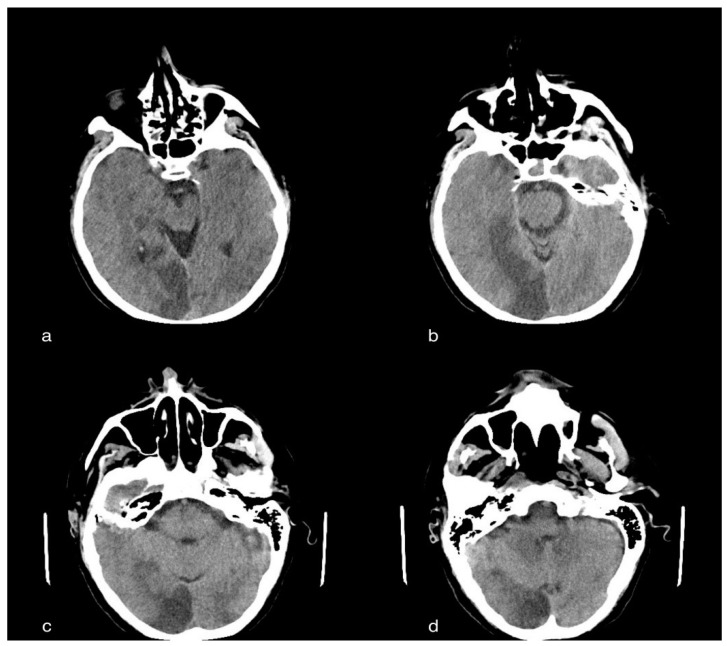
A 60-year-old male patient with acute ischemic vascular pathology in the left temporo-parietal region, cortico-subcortical in origin, extending to the ipsilateral deep white matter, characterized by a slight parenchymal hypodensity, and responsible for the modest compression of the ipsilateral ventricular, subarachnoid and parenchymal compartment, but without deviation of the septum pellucid or third ventricle to the right of the line, subarachnoid and parenchymal compartments, but in the absence of deviation of the septum pellucid or third ventricle to the right of the median line or median line or signs of intracranial hypertension.

In the cerebral CT examination, we observed acute ischemic vascular pathology in the left temporoparietal region, with the cortico-subcortical site, extending to the ipsilateral deep white matter characterized by a small parenchymal hypodensity, and responsible for modest compression on the ipsilateral ventricular, subarachnoid and parenchymal compartments of the same side, but in the absence of deviation of the pellucid septum or third ventricle to the right of the midline or signs of intracranial hypertension (Figure 2).

#### 3.2.3. Discussion

Cerebrovascular diseases have been reported as the most common neurological symptoms in COVID-19 patients. Neuroimaging studies of COVID-19 patients with cerebrovascular diseases showed subacute and acute ischemic infarct, acute hemorrhage and intracranial hemorrhage. The current understanding suggests that COVID-19 causes an excessive inflammatory response due to cytokine storm and, consequently, the pro-thrombotic state has a greater impact on the risk of ischemic stroke [20]. Therefore, this implies that severe acute respiratory syndrome coronavirus 2 (SARS-CoV-2) infection could cause ischemic stroke through a hypercoagulative state. The incidence of ischemic stroke in COVID-19 patients ranges from 0.9% to 2.7% [21,22,23]. Hypercoagulability as well as a systemic inflammatory response to the viral infection could lead to clinical manifestations of macro- and micro-thrombosis and ultimately to cerebrovascular accidents. Reports of a patient with elevated D-dimer levels strongly suggest the role of a hypercoagulable state. Patients exhibit altered levels of procoagulant factors, such as D-dimer, fibrinogen and disseminated intravascular coagulopathy [24].

Stroke signs and symptoms are:

(1) Sudden numbness or weakness of the face, or of the limbs, especially on one side of the body.
(2) Sudden confusion, difficulty speaking or difficulty understanding speech.
(3) Sudden difficulty seeing in one or both eyes.
(4) Sudden difficulty walking, dizziness, loss of balance or lack of coordination.
(5) Sudden severe headache with no known cause.

An ischemic stroke should be suspected if there is one of these symptoms associated with the alteration of the aforementioned laboratory values.

It is important that COVID-19 patients suffering from ischemic stroke undergo a full diagnostic work-up, including brain imaging. However, neurological involvement has been frequently reported with symptoms such as anosmia, dysgeusia, headache, mental confusion and seizure [25]. Moreover, acute stroke detection can be a strong prognostic marker of a poor patient outcome, being the most frequent neuroimaging finding in COVID-19 patients. The role of neuroimaging in the diagnosis of acute stroke is essential, providing crucial information for clinical decisions about treatment strategies and in order to improve outcomes of patients with acute stroke. For the study of neurological events and to differentiate and diagnose neurological phenomena, the gold standard remains MRI, but in the management of the urgency of a complication, especially in COVID-19 patients, it appears to have limits compared to CT [26].

It is important to consider brain MRI studies only for accurate diagnosis in patients with unexplained neurological symptoms in COVID-19.

Rapid and accurate diagnosis is crucial for the management of ischemic stroke. Nonenhanced CT provides high sensitivity and specificity for diagnosing ischemic stroke, ranging from 95% to 98% [27]. CT is an important clinical tool, providing accurate and timely diagnostic information that allows time-critical decision making in stroke patients in what concerns thrombolytic therapy. Non-contrast CT brain has a crucial role in the evaluation of ischemic stroke and the involvement of the major vessels.

### 3.3. Case 3: Rectus Hematoma

#### 3.3.1. Clinical History

A 75-year-old female patient presented to the respiratory emergency department with a chief complaint of dyspnea. She was complaining of severe non-productive cough, fever and fatigue for some days. The result of the RT-PCR test was positive for COVID-19. A blood investigation revealed a total leucocyte count of 15,000/μL, hemoglobin (HB) of 12 g/dL, platelet count of 444 × 103, partial thromboplastin time (PTT) of 32 s, international normalized ratio (INR) of 1.4, erythrocyte sedimentation rate (ESR) of 110 mm/1 h and C-reactive protein (CRP) of 100 mg/L.

#### 3.3.2. CT Imaging Finding

**Figure 3:**
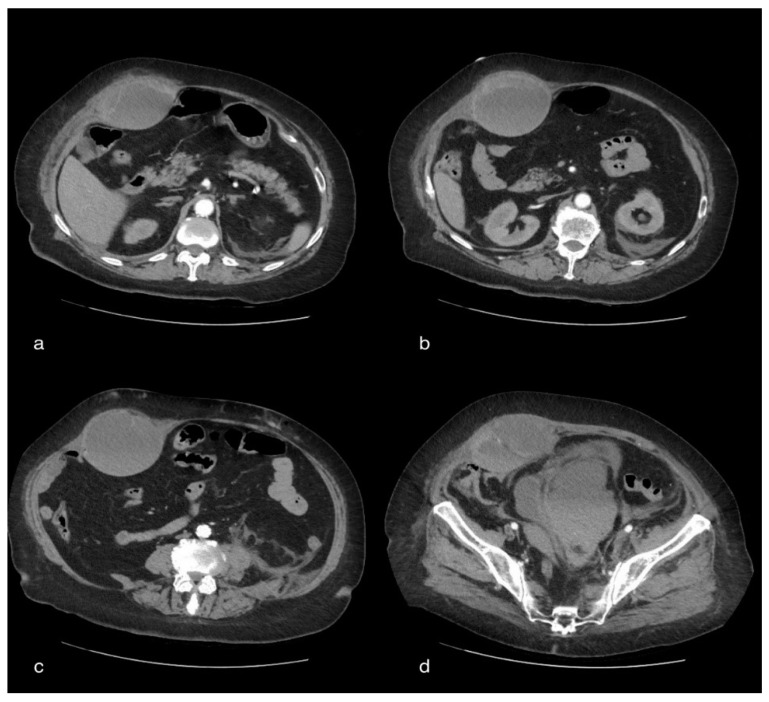
A 75-year-old female patient with volumetric increase in the rectus muscle of the right abdomen of approximately hypo/hyperdense inhomogeneous densitometry due to hematoma. The pelvic cavity appears to be occupied by a voluminous fluid/overfluid collection spontaneously hyperdense in its more declivity.

CT showed, bilaterally, the presence of multiple densitometric areoles of the GGO type with predominantly subpleural distribution with thickening of the interlobular septa with a “crazy paving” pattern. Lymphadenomegalies were observed in the mediastinum as well as ventilating phenomena. The volumetric increase in the right abdominal muscle of about 104 × 73 mm in the axial plane with a craniocaudal extension of about 28 cm, with inhomogeneous hypo/hyperdense densitometry, referred to as a hematoma, was reported. The pelvic excavation appeared to be occupied by a voluminous fluid/superfluid collection (dAP 20 cm × dcc 10 cm) spontaneously hyperdense in its most descending hematic part, appreciable in the context of two ecstatic vascular structures that were evident only in the portal and late phases of the study, compatible in the first hypothesis of venous bleeding. The uterus and the catheterized bladder appear to laterodeviate to the right. Further super-fluid collection with similar densitometric characteristics can be appreciated in the peri- and sub-renal site, posteriorly to the left with signs of hematic infarction of the ipsilateral psoas, which appears to have increased in volume. There was also a thin layer of pouring in the perihepatic area (Figure 3).

#### 3.3.3. Discussion

It is recognized that COVID-19 infection causes hypercoagulability due to an elevated level of proinflammatory cytokine, including IL-6 [28]. Moreover, the potential risk of coagulopathy is increased by elevated D-dimer and fibrinogen degradation products, which are associated with a poorer prognosis. Therefore, the treatment of the hypercoagulable state requires a proper anticoagulation therapy. Although anticoagulation therapy has beneficial effects in COVID-19 patients, it has been associated with bleeding events in the body [29]. Acute abdomen pain can uncommonly be caused by rectus sheath hematoma, which can be a life-threatening complication in COVID-19 patients receiving anticoagulation agents. It is an uncommon complication of anticoagulation therapy in COVID-19 patients. Hemodynamic instability with an increase in hematoma size was observed. When these conditions occur, a CT study with a contrast medium is highly recommended.

The clinical symptoms of rectus sheath hematoma are: nausea, vomiting, fever, abdominal and lumbar pain and abdominal tenderness on physical examination [30]. Early diagnosis and intervention are crucial in order to reduce patient mortality and morbidity. In 50% of rectus sheath hematoma cases, an evident hematoma is a useful sign. Imaging tests should be requested anyway in order to determine a differential diagnosis of rectus sheath hematoma in the case of abdominal pain. The diagnosis is confirmed by abdominal and pelvic CT scanning, which has a sensitivity of 100%. CT scan with IV contrast is deemed the gold standard for the identification of bleeding and in order to differentiate arterial and venous bleeds. A CT scan with contrast also provides information about contrast extravasation and all the intra-abdominal structures [31].

### 3.4. Case 4: Active Bleeding of the Right Lower Limb

#### 3.4.1. Clinical History

A 85-year-old female patient with a history of hypertension, obesity, diabetes and previous cholecystectomy reported fever and a non-productive cough. The result of the RT-PCR test was positive for COVID-19. She presented to the respiratory emergency department with a chief complaint of dyspnea. Blood investigation revealed a total leucocyte count of 14,800/μL (92% neutrophils), hemoglobin (HB) of 8.5 g/dL and C-reactive protein (CRP) of 50 mg/L. Blood pressure was 85/50 mmHg.

#### 3.4.2. CT Imaging Finding

**Figure 4:**
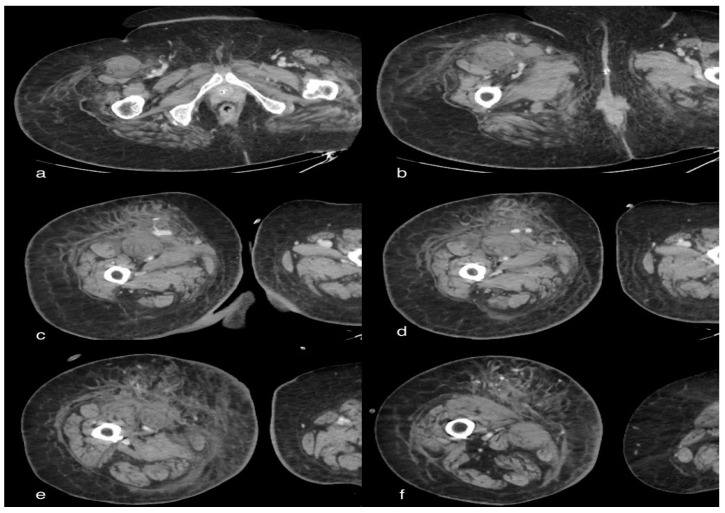
A 85-year-old female patient in whom we observe, in the late phases of the study, a marked and diffuse imbibition of the soft tissues on the antero-medial side of the right thigh due to evidence of extravasation of mdc (arterial) in correspondence of the anterior side by two collateral branches of the superficial femoral artery ipsilateral to the middle III and distal III, as well as signs of active bleeding.

The chest CT showed, bilaterally, the presence of multiple densitometric areoles of the GGO type with predominant subpleural distribution, especially in the slopes, with thickening of the interlobular septa with a “crazy paving” pattern. Three was also widespread ventilation phenomena. Pericentimetric lymphadenomegalies were observed in mediastinum. Visual Score: LSD: 25–50%; LM: 0–25%; LID: 25–50%; LSS: 25–50%; LIS: 25–50%. Abdomen CT revealed, on the anteromedial side of the right thigh, the marked and wide-spread imbibition of the soft tissues due to evidence of contrast medium (arterial) extravasation in correspondence with the anterior side by two collateral branches of the superficial femoral artery, homolateral to the middle III and the III distal as a sign of active bleeding in the lower limb (Figure 4).

#### 3.4.3. Discussion

Hyperinflammation and hypercoagulability are the systemic complications of COVID-19 [8]. Altered coagulation parameters with a considerable increase in D-dimer levels associated with severity of illness and a high risk for venous thromboembolism were identified with high prevalence. Therefore, it is important to administer antithrombotic prophylaxis in order to prevent micro- and macro-thrombi [29]. Spontaneous bleeding is characterized by an intra- or extra-muscular collection of blood, mainly located in the iliopsoas and rectus muscle because of the epigastric or femoral artery injury. It is a potentially severe complication for patients treated with anticoagulant therapy as it can lead to hemodynamic instability and consequently a life-threatening condition. Other risk factors of spontaneous bleeding include a high body mass index, arterial hypertension, comorbidities and increased abdominal pressure [32]. The clinical presentations involve severe lower abdominal pain with hemodynamic instability. A CT scan with contrast is recommended in order to confirm the diagnosis and measure the bleeding volume. Active bleeding during a CT scan is considered a warning sign requiring urgent management. CT constitutes a valuable tool to guide embolization as spasm, hypotension, tamponade and technical limits can reduce the visibility of the bleeding at angiography. Arterial blush and late contrast enhancement are two important aspects to take into account during treatment decision making because of the high sensitivity of the angio-CT (87%) [33].

### 3.5. Case 5: Renal Hematoma

#### 3.5.1. Clinical History

A 42-year-old male SARS-COVID-19 patient was admitted to hospital from home after 1 week of cough, loss of appetite, hyposomnia, asthenia and fever (40 °C). He mentioned several dyspnea for 1 day. Blood oxygen was 85%. The patient had a history of polycystic kidney disease (PKD) and no other comorbidities.

#### 3.5.2. CT Imaging Finding

CT showed volumetric increase in both kidneys, showing parenchymal structure completely subverted by the presence of multiple cystic formations, some of which were spontaneously hyperdense. In particular, on the right posterior pararenal site, there was an extended spontaneously hyperdense area indissociable from the contiguous renal parenchyma, compatible in the first hypothesis with hematoma with extension of about 120 mm and maximum axial thickness of about 35 mm. On the same side, an uneven appearance of the perirenal fat was associated with a thickening of the anterior pararenal fascia in the context of which an oval collection of 44 × 29 mm was observed below, in apparent connection with the lower pole of the kidney. There was thickening of the left posterior pararenal fascia.

**Figure 5:**
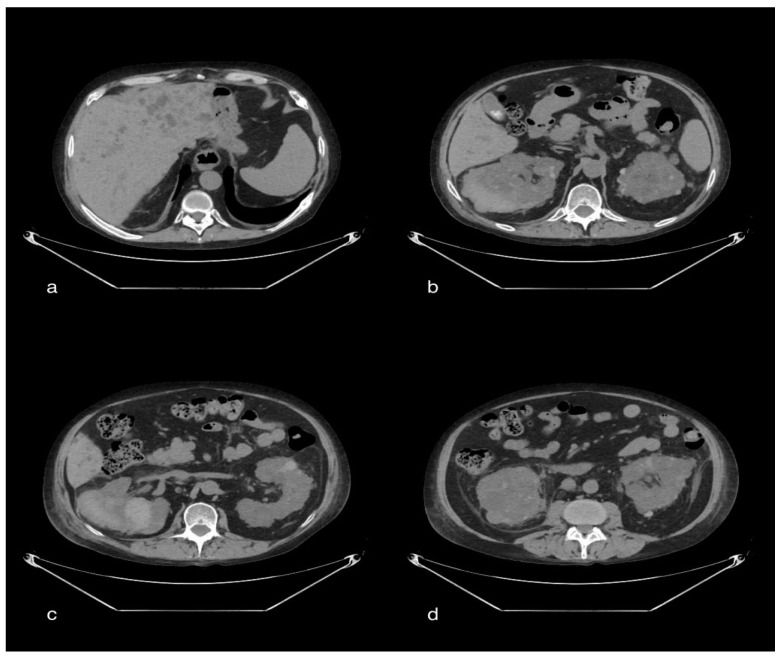
A 47-year-old male patient with multiple cystic formations in the liver and both kidneys. In particular, the kidneys show increased volume and completely subverted parenchymal structure. In the right posterior pararenal area, there is a large spontaneously hyperdense area inseparable from the adjacent renal parenchyma, which is compatible with a hematoma. On this side, there is an inhomogeneous appearance of the perirenal fat. Thickening of the left posterior pararenal fascia. Distended gallbladder, with evidence of multiple endoluminal lithiazed formations (**b**).

There was also non-free intra-abdominal effusion (Figure 5).

#### 3.5.3. Discussion

The elevation of C-reactive protein and inflammatory cytokines in COVID-19 patients cause a severe extrapulmonary and hematologic clinical presentation that can provoke hematologic complications (such as thrombosis or bleeding). Common bleeding sites in COVID-19 patients include chest wall, muscles, adrenal glands, brain, liver and retro peritoneum. The high incidence of hypercoagulation in COVID-19 patients increase the mortality rate. Antithrombotic drugs such as heparin are highly recommended. Anticoagulants are the most common cause of retroperitoneal bleeding in COVID-19 patients [34]. The clinical symptoms vary from mild persistent pain to devastating acute abdominal conditions, such as acute appendicitis or perforated viscus or dissecting aneurysm. Lenk’s triad consists of acute flank pain, tenderness and hypovolemia. Therefore, a contrast-enhanced CT scan is necessary in order to confirm the diagnosis or to rule out another diagnosis. CT scan has higher sensitivity and specificity for diagnosing renal tumor/abscess [35].

### 3.6. Case 6: Pneumomediastinum

#### 3.6.1. Clinical History

A 48-year-old old male patient exhibited no known comorbidities. He presented to the respiratory emergency department with non-productive cough, asthenia, myalgia and fever (39 °C). An oropharyngeal PCR swab was performed and tested positive for COVID-19 pneumonia. An arterial blood gas (ABG) test performed and it reported: pH 7.5, pO^2^ 56.2 mmHg, pCO^2^ 31.8 mmHg, HCO^3^-26.2 mEq/L, and Lac 2.2 mmol/L.

#### 3.6.2. CT Imaging Findings

Diffuse subcutaneous emphysema in the cervical and parietal-costal region is more anteriorly marked. There was also massive pneumomediastinum and thin anterior apical PNX flap on the right.

**Figure 6:**
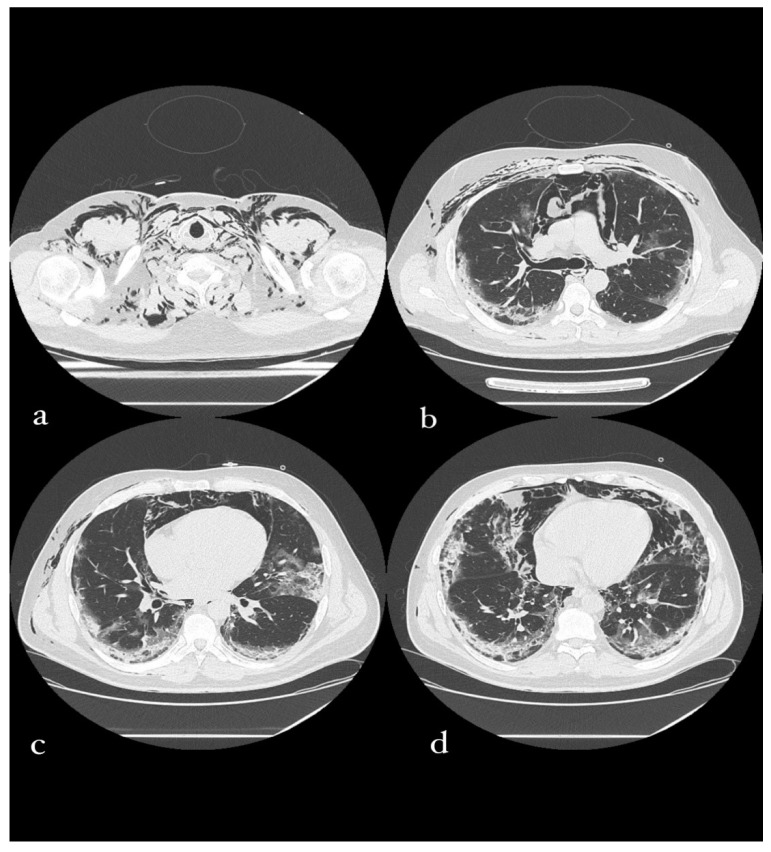
A 49-year-old male patient in whom massive invasive ventilation resulted in the formation of pneumomediastinum. The formation of pneumomediastinum is due to traumatic rupture of the alveoli (caused by invasive ventilation), a phenomenon known as the Macklin effect. Subcutaneous emphysema can also be seen.

Evidence of multiple areoles of GGO-type densitometric increase with associated thickening of the interlobular septa from crazy paving was found in both lungs, predominantly subpleural. There are multiple fibrotic striae with a course parallel to the pleura, more evident in the lower lobes. No significant ilo-mediastinal adenomegalies and no pleural effusion were reported (Figure 6).

#### 3.6.3. Discussion

A rare complication such as pneumomediastinum (PM) is observed in patients with severe coronavirus disease-19 and it is caused by alveolar rupture due to a high intra-alveolar pressure or low perivascular pressure with the well-known Macklin phenomenon. It can be spontaneous as a consequence of the intense inspiratory effort in the COVID-19-induced cytokine storm, resulting in a diffused alveolar injury, making the alveoli more susceptible of rupture. These alterations are specific to SARS-CoV-2; this may explain the higher incidence of pneumomediastinum in COVID-19 patients compared to other types of pneumonia at equal severity [36,37]. Tacconi’s study attests that the incidence of pneumomediastinum was 1.37/1000 patient-days [38]. The pathophysiology of spontaneous PM is characterized by the Macklin phenomenon, where alveolar rupture, caused by a large pressure gradient between the marginal alveoli and the lung interstitium, results in a leakage of air into the surrounding bronchovascular sheath. Moreover, the steroids used for treatment weaken the pulmonary interstitial tissue, causing alveolar air leak [39]. The clinical symptoms of PM in such patients are rapid progression or acute presentation of dyspnea, chest pain, tachycardia, tachypnea, subcutaneous emphysema and hypoxemia. Patients with these symptoms were promptly tested to rule out PM. Pulmonary complications vary from acute respiratory distress syndrome to pneumothorax. The chest CT of COVID-19 patients is characterized by ground-glass opacities and/or consolidations as well as rare findings, such as pneumo-thorax, pneumomediastinum and pneumopericardium. The reported incidence of PM in patients with ARDS is 4% [40]. PM may potentially be a prognostic marker of an adverse event that complicates the clinical course of COVID-19 patients and leads to a poor prognosis. A high mortality rate could also be linked to COVID-19 where SPM is related with a poor outcome when it occurs on a background of severe diffuse parenchymal lung damage. Imaging is crucial in the diagnosis of pneumomediastinum, given the vague nature of the clinical presentations, and should be considered in all COVID-19 patients with unexplained clinical deterioration [36]. Compared to the radiographic study CT is more useful for the detection of air in the mediastinum and for a sure diagnosis establishment or confirmation. It may also provide extra diagnostic information concerning the exclusion of comorbid conditions, such as the perforated esophagus. The exposure to very high levels of radiation is certainly its major disadvantage. Conversely, an additional advantage of CT is its possibility to reveal the Macklin phenomenon. The latter is displayed on CT scans as linear collections of air contiguous to the bronchovascular sheaths [41]. SPM is usually a self-recovering condition; however, it may require some adjustment in the management of respiratory support in order to balance adequate oxygenation and prevent pneumomediastinum expansion and pneumomediastinum-related cardiovascular and respiratory complications.

### 3.7. Case 7: Paravertebral Abscess Collection

#### 3.7.1. Clinical History

A 27-year-old male patient was admitted to the respiratory emergency department with dyspnea, back pain, hypomobility of the left lower limb and fever (39 °C). He had active pulmonary tuberculosis. The result of the RT-PCR test was positive for COVID-19. Blood investigation revealed a total leucocyte count of 12,600/μL (69% neutrophils), blood platelets (PLT) of 1256 U/mL, hemoglobin (HB) of 9.4 g/dL, lactate dehydrogenase (LDH) of 300 mU/mL and C-reactive protein (CRP) of 124 mg/L. Blood pressure was 110/80 mmHg.

#### 3.7.2. Imaging Finding

**Figure 7:**
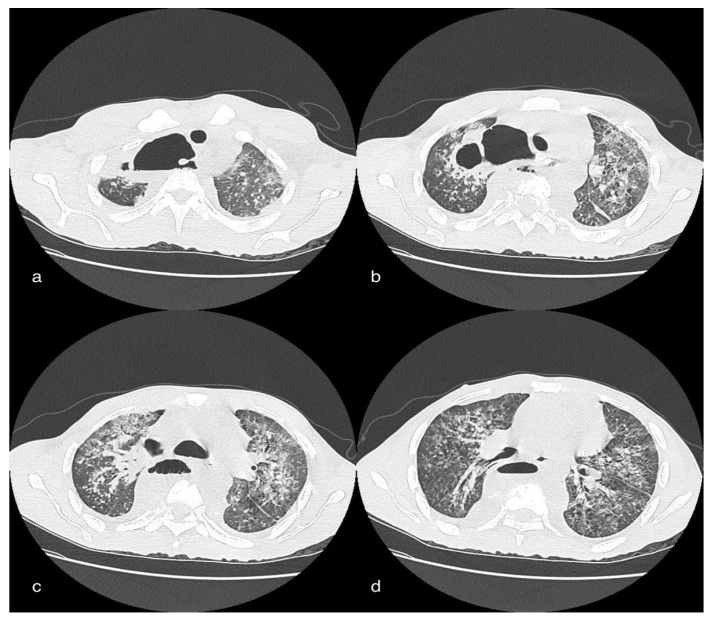
Female patient, 26 years old, of African descent in whom there is ample cavitation of two pre- and paravertebral abscess collections of the dorsal tract. The cranial collection (**a**,**b**) extends from D2 to D5 and leads to compression of the contiguous lung parenchyma, to the apical segment of the LSD, and compresses and latero-deflects the trachea to the left. From this collection, the air-borne component is also carried cranially into the right cervical prevertebral space (**b**). Another collection (**c**,**d**), with the same characteristics and with an extended aerial component, can be seen in the prevertebral space between space between D5 and D10. Evidence of an airway between the two collections at D5.

CT showed extensive cavitation of the known pre- and paravertebral abscess collection of the dorsal tract. The cranial collection extended from D2 to D5 and determined the compression of the contiguous lung parenchyma, at the apical segment of the LSD, and compressed and latero-deviated the trachea to the left. Furthermore, from this collection, the aerial component was carried cranially in the right cervical prevertebral space, up to the upper limits of the field of study. A further collection, with the same characteristics and with an extensive aerial component, was seen in the prevertebral space between D5 and D10. There was evidence of air travel between the two collections at D5. Diagnostic integration with contrast medium was required. Parenchymal alterations of the tree-in-bud type with a tendency to confluence were stable, involving almost all lung lobes, with more marked distribution at the central site. Sacked super-fluid pouring is placed in the right basal seat, with a maximum thickness of 20 mm in the anterior seat (Figure 7).

#### 3.7.3. Discussion

COVID-19 manifests symptoms of respiratory tract infections, having significant involvement in different systems. A reduction in the number of lymphocytes and monocytes with a possible impaired immune response to exogenous infective agents has been highlighted. Immunocompromised patients are also at increased risk of developing abscess and lymphopenia due to COVID-19 [34]. Bacterial and co-infections have been reported in half of the patients. The most common presenting symptoms include fever, neck swelling, sore throat, dysphagia, dyspnea, odynophagia, drooling and respiratory distress. A potentially life-threatening complication of COVID-19 is paravertebral abscesses. Therefore, early diagnosis is crucial in the management of these patients. Deep neck infections may have a higher complication rate in COVID-19-immunocompromised patients with systemic comorbidities [35]. The clinical diagnosis of abscesses can be challenging due to the non-specificity of the symptoms. CT scan plays an essential role in diagnosis and treatment decision-making strategies. CT is a helpful diagnostic tool of vertebral abscesses. However, magnetic resonance imaging has greater specificity and it is highly recommended in order to make an early diagnosis and start treatment and, consequently, have a significantly improved outcome.

## 4. Conclusions

Since the onset of the COVID-19 pandemic, the CT imaging modality, with its high sensitivity and specificity, remains the preferred imaging choice to diagnose early the different complications associated with COVID-19, such as thrombosis, ischemic stroke, infarction and pneumomediastinum, and their management, which significantly improves the outcomes.

Often, the “COVID-19 patient” is a patient of difficult management, and diagnostic imaging can highlight some situations in the pre-clinical phase to avoid worsening or further complications. The article is innovative by collecting all the possible complications observed in our experience in a single article, in order to offer the clinician and radiologist a simple checklist of the complications that the COVID-19 patient may present and indicate the case in which CT scans are the imaging method of choice.
